# Population-based analysis of the epidemiology of the surgical correction of hyperhidrosis in 1,216 patients over 11 years: a cross-sectional study

**DOI:** 10.1590/1516-3180.2021.0773.R2.14022022

**Published:** 2022-09-12

**Authors:** Marcelo Fiorelli Alexandrino da Silva, Andressa Cristina Sposato Louzada, Marcelo Passos Teivelis, Nickolas Stabellini, Dafne Braga Diamante Leiderman, José Ribas Milanez de Campos, Edson Amaro, Nelson Wolosker

**Affiliations:** I MD. Attending Physician, Department of Vascular and Endovascular Surgery, Hospital Israelita Albert Einstein, São Paulo (SP), Brazil.; II MD. Research Fellow, Department of Vascular and Endovascular Surgery, Hospital Israelita Albert Einstein, São Paulo (SP), Brazil.; III MD, PhD. Attending Professor, Faculdade Israelita de Ciências da Saúde Albert Einstein (FICSAE), Hospital Israelita Albert Einstein, São Paulo (SP), Brazil; IV Undergraduate Medical Student, Faculdade Israelita de Ciências da Saúde Albert Einstein (FICSAE), Hospital Israelita Albert Einstein, São Paulo (SP), Brazil.; V MD, PhD. Attending Physician, Department of Vascular and Endovascular Surgery, Hospital Israelita Albert Einstein, São Paulo (SP), Brazil.; VI MD, PhD. Associate Professor, Department of Surgery, Faculdade de Medicina, Universidade de São Paulo (USP), São Paulo (SP), Brazil; VII MD, PhD. Associate Professor, Department of Radiology, Faculdade de Medicina, Universidade de São Paulo (USP), São Paulo (SP), Brazil; VIII MD, PhD. Full Professor, Faculdade Israelita de Ciências da Saúde Albert Einstein (FICSAE), Hospital Israelita Albert Einstein, São Paulo (SP), Brazil;

**Keywords:** Sympathectomy, Big data, Public health, Endoscopic thoracic sympathectomy, Video-assisted thoracic surgery, Oxybutynin

## Abstract

**BACKGROUND:**

Endoscopic thoracic sympathectomy is the definitive surgical treatment for hyperhidrosis and a nationwide study has suggested that cultural and socioeconomic factors play a role in the numbers of operations performed. Thus, there is a need to evaluate local data in order to understand the local epidemiology and trends in hyperhidrosis treatment.

**OBJECTIVE:**

To study the epidemiology of sympathectomy for treating hyperhidrosis in São Paulo, the largest city in Brazil.

**DESIGN AND SETTING:**

Population-based retrospective cross-sectional study.

**METHODS:**

Data on sympathectomies for treating hyperhidrosis between 2008 and 2018 were assessed from the database of the Municipal Health Department of São Paulo, Brazil.

**RESULTS:**

65.29% of the patients were female, 66.2% were aged between 20 and 39 years and 37.59% had registered with addresses outside São Paulo. 1,216 procedures were performed in the city of São Paulo from 2008 to 2018, and 78.45% of them were in only two public hospitals. The number of procedures significantly declined over the years (P = 0.001). 71.63% of the procedures were associated with 2-3 days of hospital stay, only 78 intensive care unit days were billed and we did not observe any intra-hospital death.

**CONCLUSION:**

The profile of patients operated on in São Paulo (young women) is similar to that described in other populations. Sympathectomy is a very safe procedure, with no mortality in our series. There was a decreasing trend in the number of surgeries over the years.

## INTRODUCTION

Sweat exceeding the needs of thermoregulation in certain body areas, due to hyperfunctioning sweat glands, characterizes hyperhidrosis (HH). This condition seems to be related to higher levels of cholinergic acetylcholine and nicotinic alpha-7 receptors in these individuals’ sympathetic ganglia.^1^ This disease, which affects up to 2.8%-4.8% of the population,^2,3^ has a significant negative impact on patients’ quality of life, and it impairs their personal and professional relationships.^4^ HH commonly begins in childhood and can persist throughout adulthood if not properly treated.^2,5^

Currently, the definitive surgical treatment for HH consists of endoscopic thoracic sympathectomy (ETS), which is safe and clinically effective and results in significant improvement in quality of life.^4,6,7^

Although this treatment is covered by the Brazilian National Health System (Sistema Único de Saúde, SUS), a recent nationwide analysis reported that there was large variability in terms of regional ETS rates for treating HH that could not be fully explained by climate differences. Thus, it was suggested that cultural and socioeconomic factors have contributed to the numbers of ETS performed.^8^ Hence, we hypothesized that in order to learn the local epidemiology and trends in HH treatment, it may be necessary to evaluate local data as well.

Therefore, we designed the present study to assess the epidemiology and outcomes of ETS for treating HH in the city of São Paulo, which is the largest, wealthiest and most populous city in Brazil, with an estimated population of 12 million inhabitants,^9^ among whom more than 5 million are exclusively dependent on SUS.^10^ Furthermore, the database of the Municipal Health Department of São Paulo provides the most detailed health data,^11^ thus yielding more information than the national database.

## OBJECTIVE

To study the epidemiology and outcomes of ETS for treating HH in the city of São Paulo, with evaluation of the total number of ETS performed for treating HH, hospital volumes, trends over time, costs, in-hospital mortality and lengths of hospital and intensive care unit (ICU) stay, along with the proportion of patients from other cities who came to São Paulo to undergo ETS for treating HH.

## METHODS

This study involved analysis of data available on the health information (Informações em Saúde, TabNet) platform of the Information Technology Department of SUS (Departamento de Informática do Sistema Único de Saúde, DATASUS),^11^ which provides open data on procedures performed in accredited public hospitals. Such accreditation is a prerequisite for government reimbursement for the surgeries performed.

Data regarding sympathectomies for treating HH (as coded in accordance with the International Classification of Diseases, 10^th^ edition: R61) covering the years 2008 to 2018 were selected from TabNet of the Municipal Health Department of São Paulo. Among the selections, sex, municipality of residence, age group, number of surgeries performed (total and per establishment), mortality during hospitalization, length of stay in the establishment, ICU stay and amounts paid were analyzed.

Age was stratified as follows: under 14 years, between 15 and 19 years, between 20 and 39 years and over 39 years. The amounts paid in Brazilian official currency (reais, R$) were converted into United States dollars (USD) on December 31, 2012, which was the midpoint date between the first and last data analyzed, at the rate of 1.00 USD = R$ 2.04). Hospitals were numbered, in descending order, according to the total number of procedures performed.

The information was obtained from publicly accessible websites by means of computer programs for accessing content from web scraping codes. These codes were programmed in Python language, version 2.7.13 (Python, Beaverton, Oregon, United States), in the Windows 10 single-language operating system. The steps of collecting and selecting fields on the platform and later adjusting the tables were performed using the Selenium WebDriver package, version 3.1.8 (Selenium HQ, various contributors around the world); and Pandas, version 2.7.13 (Lambda Foundry, Inc., and PyData Development Team, New York, United States). We used the Mozilla Firefox browser, version 59.0.2 (Mountain, California, United States), and the Geckodriver WebDriver. version 0.18.0 (Mozilla Corporation, Bournemouth, England).

After collection and treatment, the data were organized and grouped in a spreadsheet in the Microsoft Office Excel 2016 program, version 16.0.4456.1003 (Microsoft, Redmond, Washington, United States).

For statistical analysis, linear regression analysis was performed to evaluate the trends in procedures over the years, using the SPSS version 22.0 (IBM Corp. Armonk, New York, United States). For all tests, the level of statistical significance was taken to be α = 0.05.

This study was approved by the ethics committee of the institution where it was conducted (procedural number 3067-17; approved on July 18, 2017). Data are anonymous at DATASUS; therefore a waiver of informed consent was requested and granted by our institutional review board.

## RESULTS

Most of the patients were female (65.29%), aged between 20 and 39 years (66.2%) and with a registered residential address in the city of São Paulo (62.41%). The age stratification of the operated patients is presented in Table 1.

**Table 1.** Age stratification into four subgroups (< 14 years, 15–19 years, 20–39 years and > 39 years)

Age	n	%
< 14 years	75	6.16
15–19 years	251	20.64
20–39 years	805	66.2
> 39 years	85	6.99

**Figure 1.**
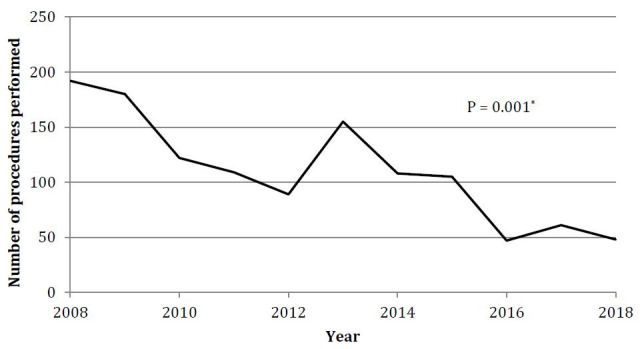
Distribution of sympathectomies for treating hyperhidrosis between 2008 and 2018, in public hospitals in São Paulo.

In total, 1216 procedures were performed in the city of São Paulo from 2008 to 2018. Most surgeries (78.45%) were performed in only two public hospitals. The distribution of procedures in each hospital and the overall distribution of procedures over the years are shown in Table 2 and Figure 1, respectively.

**Table 2.** Number of procedures performed in each hospital between 2008 and 2018

Hospital	2008	2009	2010	2011	2012	2013	2014	2015	2016	2017	2018
1	153	158	81	65	32	94	45	36	34	31	24
2	3	0	17	12	20	34	42	53	3	10	7
3	28	9	13	10	3	12	0	0	0	0	1
4	2	0	0	0	11	7	9	7	3	16	11
5	2	7	3	10	18	7	5	0	3	1	0
6	3	3	2	4	1	0	1	0	0	1	1
7	3	0	0	2	0	1	1	3	0	0	1
8	0	1	1	4	1	0	3	1	0	0	0
9	0	0	0	0	0	0	0	5	3	0	1
10	0	0	0	2	2	0	1	0	0	2	2
11	3	1	4	0	0	0	0	0	0	0	0
12	2	1	0	0	1	0	0	0	1	0	0
13	0	0	0	0	0	0	1	0	0	0	0
14	0	0	1	0	0	0	0	0	0	0	0

*Linear regression analysis to assess the trend of reduction in the number of procedures over the years.

We observed a progressively decreasing trend in the total number of procedures over the years (P = 0.001). The first year studied, 2008, presented the largest number of cases (192 surgeries), whereas in recent years there have been fewer cases, especially in 2016 (47 surgeries).

In all the years evaluated, we did not observe any case of intra-hospital death. In other words, no patient died within the same hospital admission following sympathectomy for hyperhidrosis in the city of São Paulo during the period studied.

**Figure 2.**
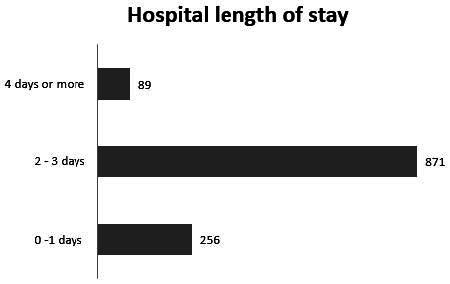
Distribution of patients according to length of hospital stay in days.

The number of procedures classified according to length of hospital stay is shown in Figure 2. We observed that procedures with a length of hospital stay of 2 to 3 days predominated (71.63%). Only 89 patients (7.31%) remained in the hospital for 4 days or more.

Regarding ICU stay, SUS reimbursed a total of 78 days of ICU stay for the entire cohort: 2 days of ICU were paid for in 2008, 24 days of ICU in 2010, 2 days of ICU in 2015 and 50 days of ICU in 2017. In all other years, no ICU stay was recorded for any patient.

The number of procedures, equivalent dollar amounts reimbursed by SUS and average amounts per procedure according to hospital establishment are presented in Table 3.

**Table 3.** Number of procedures (%), amounts reimbursed by the public healthcare system and average amount per procedure, in United States dollars

Hospital	Number of procedures (%)	Total reimbursed	Average amount per procedure
1	753 (61.92)	314,153.19	417.20
2	201 (16.53)	94,278.25	469.04
3	69 (5.67)	32,493.74	470.92
4	66 (5.43)	33,052.79	500.80
5	56 (4.61)	26,898.84	480.34
6	16 (1.32)	7,610.58	475.66
7	11 (0.90)	5,411.38	491.95
8	11 (0.90)	4,607.50	418.86
9	9 (0.74)	5,467.13	607.46
10	9 (0.74)	4,209.31	467.70
11	8 (0.66)	3,293.05	411.63
12	5 (0.41)	2,336.79	467.36
13	1 (0.08)	658.72	658.72
14	1 (0.08)	445.67	445.67
**Total**	**1,216 (100)**	**534,916.96**	**439.90**

A total of 534,916.96 USD was reimbursed by SUS for all the surgeries, corresponding to an average amount of 439.90 USD per surgery. The reimbursement per procedure ranged from 417.20 USD to a maximum of 607.46 USD over the years.

## DISCUSSION

### Patients’ demographics

In line with previous reports, we observed a predominance of female patients.^1,8^ Even though the prevalence of HH is similar between the sexes,^5^ there is a greater demand for treatment among females, likely due to generally greater esthetic concern in this group.^12,13,14,15^

Regarding age, ETS for treating HH has proven to be beneficial for a wide range of age groups.^16,17^ However, the demand for treatment of hyperhidrosis is estimated to be greater in young and economically active age groups,^5^ which is in agreement with our findings, as most individuals in our study were aged between 20 and 39 years (66.2%).

More than one third of the patients had registered with addresses outside of the city of São Paulo (37.59%). Healthcare inequalities in Brazil and the concentration of resources in its southeastern region, and in some southeastern centers like the city of São Paulo, are well documented.^9^ A similar high proportion of out-of-town patients who come to the city of São Paulo to seek treatment has been observed in other reports.^18^

The state of São Paulo comprises 645 municipalities, which are organized into 17 healthcare service reference regions (HSRR).^19^ Ideally, in accordance with the principle of regionalization of the Brazilian National Health System, each HSRR should be designed to meet most of the healthcare demands of its municipalities.^20^ The city of São Paulo is the only municipality included in its HSRR.^19^ Hence, the patients treated by mean of sympathectomy for hyperhidrosis with a registered address outside of the city of São Paulo, belonged to other HSRRs.^19^ It is likely that many of these patients went to São Paulo to seek treatment, thus practicing healthcare tourism, instead of being properly referred from their own municipality to a center in the main city of their own HSRR. Being aware of these statistics is important, from the point of view of better allocation of resources and regulation of healthcare demands, in order avoid overloading the healthcare services and incurring deficits in the revenue of the healthcare system.

### Procedure rates and trends

Starting in the first year of the period studied, we observed a progressively downward trend in the number of ETS procedures for treating HH. This contrasts with national statistics showing an initial upward trend between 2008 and 2012, and a downward trend thereafter.^8^ This difference may have been because the municipal center with the highest volume of ETS (hospital 1) was also the one in which an “oxybutynin first” protocol for treating HH started to be used in 2007, such that sympathectomy was then reserved for refractory cases or for patients with intolerance to oral treatment, in an attempt to reduce the incidence of compensatory hyperhidrosis, a common complication after sympathectomy.^21,22,23,24^ Good results from the “oxybutynin first” strategy started to be reported from 2011 onwards,^25,26,27,28,29,30,31^ which might well have contributed to the national decrease in use of ETS for treating HH from 2012 onwards.

### Hospital and ICU length of stay and in-hospital mortality

We observed that the hospital stay was short, in line with other studies.^32,33^ Most of the patients were discharged on the second or third day of hospitalization, which likely corresponded to the first and second postoperative days, respectively, given that patients are usually admitted on the day before the surgery.

Regarding ICU length of stay, due to the anonymity of the data, we only had access to the total number of ICU days paid by the government. Thus, we did not know how many patients were admitted to the ICU and for how many days, or what the indications for ICU admittance were. These therefore were limitations of our study. In total, we observed that 78 days of ICU stay were reimbursed to the hospitals, which were unevenly distributed over the years of 2008, 2010, 2015 and 2017 only. This number is small, but not negligible, especially when considering that most of the patients were young adults. Some of these days in the ICU may have related to patients with severe early postoperative complications, such as pneumothorax or hemothorax, which have been reported by other authors.^32^ Nevertheless, no in-hospital death subsequent to ETS for treating HH was reported in the public hospitals of São Paulo between 2008 and 2018, as reported in other large studies,^32,33^ which emphasizes the low mortality associated with this treatment.

Within the city of São Paulo, 78.45% of the procedures were performed in only two public hospitals. Considering that hyperhidrosis is a bothersome but not lethal disease, and given the underfunding of the public healthcare system,^34^ there are few referral centers dedicated to this treatment. This concentration in just a few centers would tend to improve perioperative outcomes and may likely explain the absence of fatalities.

### Costs

A total of 534,916.96 USD was reimbursed by the government for ETS for treating HH, with an average amount of 439.90 USD per procedure, a little less than reported by other authors.^35^ One of the limitations of our cost analysis was that the reimbursement was based on a compensation table for procedures, which often does not reflect the actual amount spent on procedures by the hospitals. We may conjecture that the decrease in the number of surgeries may have led to higher public expenditure on clinical treatments. As HH is a frequent disease among young people, who may use medications over the long term, this should be considered within publicly funded healthcare, although a cost-effectiveness study would be necessary in order to evaluate it properly.

### Limitations

Besides the limitations already mentioned, i.e. anonymous data precluding follow-up and adjusted analysis, along with reimbursement based on a fixed compensation table, another limitation of our study was that the database only provides data relating to selected procedures performed in accredited public hospitals. Thus, the database is susceptible to some loss of data.

On the other hand, this was a comprehensive and detailed epidemiological analysis on sympathectomy for treating HH that made use of objective data that had been compulsorily recorded in a public database. Our findings revealed the demographics of patients who were seeking surgical treatment for hyperhidrosis and the high volume of out-of-town patients who were seeking treatment in São Paulo; and highlight the safety of this treatment.

## CONCLUSIONS

Over the last 11 years, sympathectomy for treating HH has been widely performed in the city of São Paulo. The profile of patients operated on in São Paulo (young women) is similar to that described in other populations. Sympathectomy is a very safe procedure, with no mortality in our series. There was a decreasing trend in the number of surgeries over the years.
